# Tuning the Optical Band Gap of Semiconductor Nanocomposites—A Case Study with ZnS/Carbon

**DOI:** 10.3390/ma13184162

**Published:** 2020-09-18

**Authors:** Dominik Voigt, Larry Sarpong, Michael Bredol

**Affiliations:** Department of Chemical Engineering, FH Münster University of Applied Sciences, 48565 Steinfurt, Germany; dv009200@fh-muenster.de (D.V.); ls035372@fh-muenster.de (L.S.)

**Keywords:** optical band gap, band edge, quantum dot, nanocomposite, DFT

## Abstract

The linear photochemical response of materials depends on two critical parameters: the size of the optical band gap determines the onset of optical excitation, whereas the absolute energetic positions of the band edges define the reductive or oxidative character of photo-generated electrons and holes. Tuning these characteristics is necessary for many potential applications and can be achieved through changes in the bulk composition or particle size, adjustment of the surface chemistry or the application of electrostatic fields. In this contribution the influence of surface chemistry and fields is investigated systematically with the help of standard DFT calculations for a typical case, namely composites prepared from ZnS quantum dots and functionalized carbon nanotubes. After comparing results with existing qualitative and quantitative experimental data, it is shown conclusively, that the details of the surface chemistry (especially defects) in combination with electrostatic fields have the largest influence. In conclusion, the development of novel or improved photoresponsive materials therefore will have to integrate a careful analysis of the interplay between surface chemistry, surface charges and interaction with the material environment or substrate.

## 1. Introduction

### 1.1. Band Positions and Electrical Fields

**Figure 1:**
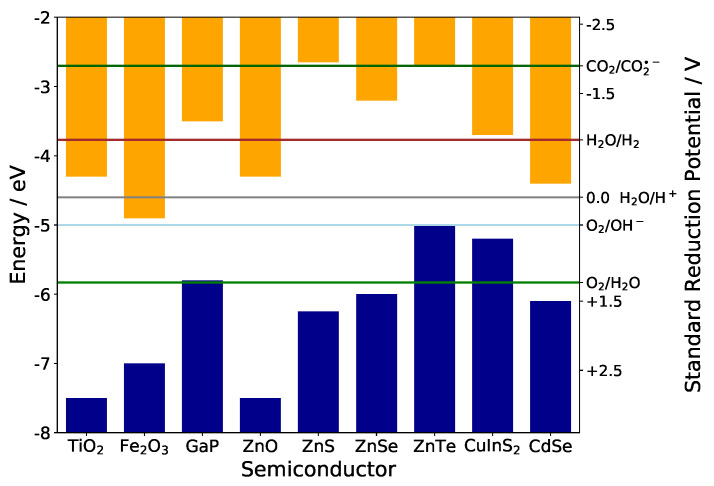
Band edge positions extracted from literature data [2,3,4,5] compared to Standard Reduction Potentials of some important redox couples.

Nanomaterials under confinement conditions show a variable band gap that can be fine tuned by careful control of particle sizes. For many applications in photovoltaics, electrochemistry, electrocatalysis or photoelectrochemistry however, the band gap value alone is not the only parameter of interest-the absolute positions of valence and conduction band edges relative to vacuum energy are defining the oxidative or reductive power of holes and electrons, respectively. Phenomena like electrocatalysis or photocatalysis therefore can only be understood in detail, when these crucial data are available-either experimentally or computationally. Figure 1 illustrates this for some typical semiconductors and some redox couples of technical interest. Very obviously the oxidative power of holes in the valence band and of electrons in the conduction band varies strongly between different semiconductors-their redox activity thus can be fine tuned by fine tuning of the band edges, if possible. As a rule of thumb, after optical excitation many oxides are strong oxidizers at their particle surface, whereas strong reducers are rather found among the chalcogenides and pnictides. In case of strong confinement in nanoparticles the band edge positions do not necessarily shift away from their bulk positions symmetrically on the energy axis-this effect can for instance be exploited for enabling charge transfer in hybrid photovoltaics [1].

Energy levels of electrons and holes are not only depending on the chemical nature of the host crystal and its band structure, but also on the presence of electrical fields. In composites, such fields can develop at the contact between different phases (known as “band-bending” due to local imbalance in charge neutrality at the interface), or by electrostatic charges adsorbed to one or more components of the composite. In case such fields are strong enough, they are expected to alter the absolute energy levels of band edges and thus influence optical and redox behaviour. Fields originating from defects may even limit the linewidth of excitonic emission [6]. In molecular systems these effects are well documented and have been analyzed also theoretically [7]. Since most composites are applied and used inside a matrix, the interaction strength is depending strongly on the effective dielectric properties of the environment, which in many cases is containing water and therefore will be a complex ensemble of solids (with typical relative permittivities on the order of 10 or below like for ZnS) and liquids or even air. Water and other solvent molecules with orientable permanent dipoles may screen electrical fields effectively, depending on the number of mobile carriers (ions) in between the partners in a composite. These screening effects have been described at least qualitatively by the well known*Debye-Hückel*–approach which thus may provide a guideline for the effects discussed in this article. We may follow the treatment outlined in the textbook of Hiemenz [8], which leads to quasi-analytical results for charge or field screening depending on the geometry of the charged object. For the typical case of a spherical quantum dot (QD) in contact or close neighbourhood with a cylindrical, a spherical or a flat charged object, the distance (*r*)–dependent screening factor S(r) can approximately be expressed either with exponential functions or the help of zero- and first-order modified *Bessel* functions $K_{i}$ of the second kind:

(1) $$S^{\mathrm{cylinder}}\left(r\right)=\frac{K_{0}\left(r/r_{D}\right)}{K_{1}\left(R_{c}/r_{D}\right)}$$

(2) $$S^{\mathrm{sphere}}\left(r\right)=\frac{R_{s}}{r}e^{-\left(r-R_{s}\right)/r_{D}}$$

(3) $$S^{\mathrm{flat}}\left(r\right)=e^{-r/r_{D}},$$

where $r_{D}$ is *Debye*’s length, $R_{c}$ is the radius of the charged cylinder and $R_{s}$ is the radius of the charged sphere.

**Figure 2:**
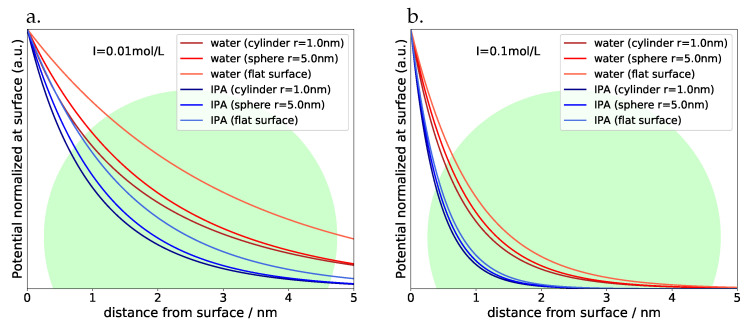
Potential screening at room temperature depending on geometry and nature of solvent (IPA: isopropyl alcohol) at (**a**) ionic strength of 0.01 mol/L (**b**) ionic strength of 0.1 mol/L.

Figure 2 shows plots of S(r) with water and isopropyl alcohole (IPA) as examples of technically interesting solvents for all three geometries and ionic strengths of 0.01 mol/L and 0.1 mol/L at room temperature (with *S* being normalized to the value at the surface of the respective object). A QD with approximately 5 nm diameter is visible for comparison as well, and we can see immediately, that for both solvents the electrical field is stretching far out into the full volume of the QD also at higher ionic strengths, whereas the details obviously depend on the interface chemistry: in the presence of larger (e.g., polymeric) ligands, the distance between dot and substrate may be very much larger than in cases, where there are no or very small ligands. We may therefore expect optical effects simply depending on the effective distance between QD and substrate. For objects of non-spherical or non-cylindrical shape we will gradually approach the situation of a QD in contact with a flat surface; as a rule, the electrostatic field over a flat surface will stretch farer out into space simply for geometrical reasons and thus lead to even stronger optical and redox effects. With increasing particle size of the semiconductor however, bulk conditions will dominate step by step–the penetration depth of electrical fields does not exceed 5 nm under most practical conditions.

### 1.2. Semiconductor Composites

**Figure 3:**
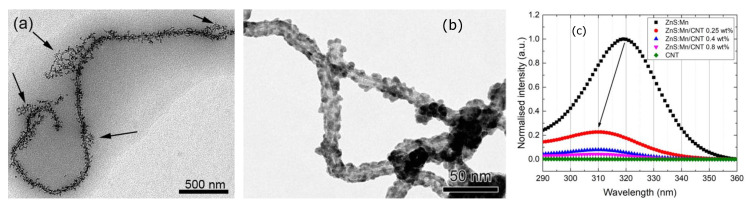
Nanocomposites prepared by heteroaggregation (**a**) or direct growth (**b**), spectral excitation shift after direct growth (**c**). All images reprinted from references [10,11] with permission from *Elsevier*.

A typical case of the conditions described above are nanocomposites formed from ligand-stabilized semiconductor QDs and a functionalized carbon support: depending on the specific local arrangements and environments spectral shifts in absorption (and excitonic emission) have been observed. They have been detected in emission as a*Stark* shift of the excitonic luminescent state in a CdSe/ZnS core shell QD conjugated to electrostatically charged carbon nanotubes (CNTs) [9] or more directly in the excitation spectra of pure ZnS nanoparticles conjugated to oxidized CNTs [10]. In the latter work an attempt was made to control the distance between quantum dots and charged support by the synthetic protocol applied: the composites were either made by electrostatically controlled heteroaggregation (leading to a more distant and loose arrangement due to the presence of ligand and solvation shells and with a morphology depending on ratios of positively and negatively charged components [11]) or by direct growth of the quantum dots on the support, leading to a more intimate connection [10]. It turned out, that only in the second case a spectral shift in the excitation spectrum could be observed, pointing to the delicate influence of the distance between dot and support. Figure 3 shows the different arrangements in the composites and the spectral shift observed (ca. 10 nm).

In most experiments with functionalized CNTs the multi-walled variants (MWCNT) are used. Due to their radius of several nanometers, they present an intermediate case between a flat substrate and a cylindical substrate. Technical graphitized carbon particles on the other hand are presenting quite rugged surfaces with local curvatures similar to MWCNTs. In Reference [10] the authors therefore did check, whether MWCNTs and a commercial carbon material (*Vulcan* from *Cabot*) did show similar effects after functionalization and combination with ZnS QDs. They did find the same optical signature in both kinds of composite. Changing the particle size of the QDs did change the excitation spectrum independently, so the shifts observed are definitely due to local electrostatic fields.

Direct experimental investigation of the positions of band edges relative to vacuum level is much more demanding than the extraction of optical band gaps for example, from excitation spectra [12]. This contribution therefore reports on the modelling of band edges and related defect states in order to be able to correlate experimental findings from optical spectra with variations in preparation and surface chemistry. Since many appplications call for semiconductors with optical band gaps in the visible or near infrared region of the optical spectrum, chalcogenides other than oxides are often in the center of interest (mostly sulfides or selenides) [13]. For surface passivation and stabilization nanoparticles of such semiconductors often are coated with a thin ZnS shell. The typical situation in any (photo-)electrochemical application therefore is an interface between ZnS and carbon in one of its graphite-like morphologies. Calculations in this contribution thus will deal with ZnS and its nanocomposites with CNTs, potentially guiding fresh experimental approaches to tuning both the band gap and the absolute positions of the band edges. Surface terminations and near-surface defects will be of special interest, since it is known from work on WO${}_{3}$, that the energy level alignment on the absolute energy scale may change drastically in defective variants [14]. Whether surface defects lead to individual traps or rather a band shift, depends on their density and therefore often is a matter of some debate. Careful computational analysis can help here, as demonstrated for example, for the case of small CdSe clusters [15]. The dominant role of surface defects is also of huge interest in lead halide perovskites, since their optical properties appear to be very defect-tolerant but nevertheless show large differences between defects in the bulk and the surface [16].

In internally structured quantum dots control of band gap and alignment of band edges has made huge progress in the last years, allowing for instance the use of defects for broad band luminescence by internal diffusion processes [17] or electrical pumping of quantum dots by using graded so-called “giant” shells around CdSe cores [18]. Thick graded shells with proper alignment are also key to replace the near-perfect but toxic CdSe cores by environmentally more benign materials like InP [19], but it was also pointed out, that the control of surface defects as traps for electrons as well as holes is key to high efficiency [20]. The most general case in this context are ternary or quaternary semiconductors, which often allow tuning band edges simply by adjustment of the stoichiometry [21], but still need protective shells like the ZnS that is used in this study as proxy for other materials.

In electrocatalysis and photocatalysis the semiconductors are interfaced with liquid phases, and the details of the catalytic processes depend on flat band potentials inside the catalyst, bent potentials at the interfaces and the presence of adsorption sites and ligands at the active interface. With the now broad availability of computational packages on the DFT level of theory, and using plane wave basis sets and pseudopotentials, these interfaces and their energetics in principle can be modelled and their properties be understood or even predicted, see for instance a recent example on the nature of the GaP electrode surface under CO${}_{2}$ reduction conditions [22]. In a current review however it has been pointed out, that the theoretical approaches still have their limitations in terms of accuracy, and therefore we will always need critical comparison with experimental data [23]. Such data are often available from electrochemical measurements, but mostly relative to the Standard Hydrogen Electrode (SHE), whereas computational data are reported relative to the vacuum level at infinite distance ($E_{\mathrm{vac}}^{\infty }$). The debate about the absolute potential of the SHE has been settled some time ago to a generally accepted value of −4.6 eV [24], which consequently will be used in this report. One has to be careful however not to confuse $E_{\mathrm{vac}}^{\infty }$ with the vacuum level just outside the solid which will be influenced by surface charges and surface dipoles [25]. Some of the uncertainty in literature reports is caused exactly by this difference.

Based on the points raised above, in this work ZnS bulk and ZnS nanoparticle properties have been evaluated by standard DFT methods, first in their pure form, and then under the influence of an electrical field, after introducing surface defects and in contact with a functionalized (charged) CNT. To avoid prohibitive computational cost, the ZnS nanoparticles evaluated are kept very small, and the CNTs are modelled in their single-walled variant. The results finally are discussed in the light of the experimental findings described above.

## 2. Results and Discussion

### 2.1. Pure ZnS and CNTs

To investigate the electronic structures and properties theoretical ab initio calculations have been performed based on density functional theory (DFT). Plane wave (PW) basis sets, ultrasoft pseudopotentials (USPP) and the Perdew-Burke-Ernzerhof (PBE) exchange-correlation functional within the generalized gradient approximation (GGA) were used to solve the Kohn-Sham equations. To improve the accuracy of these calculations and to make them comparable to experimental results the strong on-site Coulomb interaction of localized electrons, which is insufficiently described by LDA or GGA is corrected by an additional Hubbard-like term (Hubbard U parameter). For an in depth explanation on all details and the used computational methodologies see the Section 3.2.

**Figure 4:**
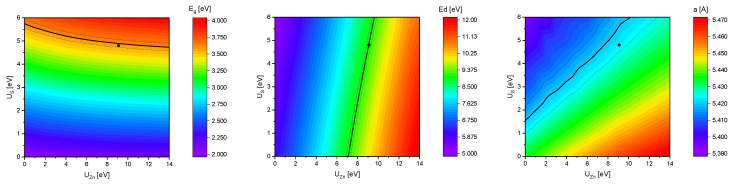
Trend of the band gap $E_{g}$ (**left**), the difference of the valence band maximum and the Zn 3d states Ed (**middle**) and the lattice parameter *a* (**right**) with respect to the U_Zn_ and U_S_ parameter.

The influence of the Hubbard U parameters U_Zn_ and U_S_ on the band gap $E_{g}$, the difference of the valence band maximum (VBM) and the Zn 3d states Ed and the lattice parameter *a* of bulk sphalerite structure ZnS (s-ZnS) is shown in Figure 4. The black solid lines are corresponding to the experimental values of $E_{g}$ = 3.6 eV [26], Ed = 9.1 eV [27] (averaged value, for the exact spin orbit splitting into $Ed_{3/2}$ and $Ed_{5/2}$ see Table 1) and *a* = 5.42 Å [28]. Since these three lines do not intersect in exactly one point a reasonable uncertainty of ± 0.05 eV (for $E_{g}$), ± 0.10 eV (for Ed) and ± 0.005 Å (for *a*) was added (dashed lines). Thus values for U_Zn_ = 9.1 eV and U_S_ = 4.8 eV were obtained that reproduced the experimental values in good agreement. The improvement from the standard GGA calculations towards more accurate results with GGA+U calculations is also shown in Table 1.

**Figure 5:**
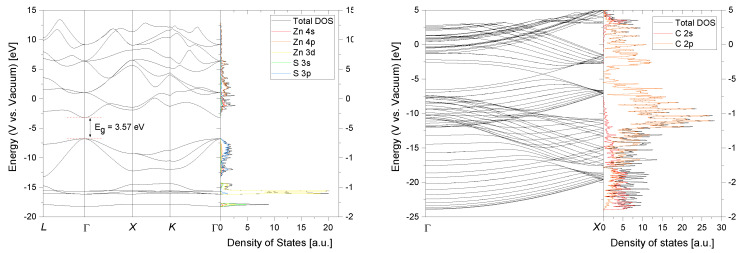
Band structure and orbital projected density of states of s-ZnS (**left**) and a 10,10 carbon nanotube (CNT) (**right**).

In Figure 5 the calculated band structure and orbital projected density of states (PDOS) is shown for bulk s-ZnS and a metallic 10,10 CNT. The absolute band energies are aligned with respect to the vacuum energy. The energy of the valence band maximum (VBM), conduction band minimum (CBM) and the Fermi energy correspond therefore physically to the ionization potential (IP), electron affinity (EA) and work function (W). These crucial properties are surface orientation dependent and are calculated for the s-ZnS (110) surface since it is the thermodynamic most stable one with the lowest surface energy [30]. It should be mentioned that these values varied vastly with respect to the termination and orientation of the surface (see Figure A2). The obtained results were EA_110_ = 3.13 eV and IP_110_ = 6.70 eV which corresponds to a error of about ∼0.8 eV when comparing to the experimental values (see Table 1) and should be considered when interpreting the following results. Nevertheless, it is still in good agreement with what is achievable by first-principles calculations [31]. The work function of the 10,10 CNT is W = 4.50 eV which is in good agreement to experimentally determined values for single walled carbon nanotubes (SWCNT) [32,33].

**Figure 6:**
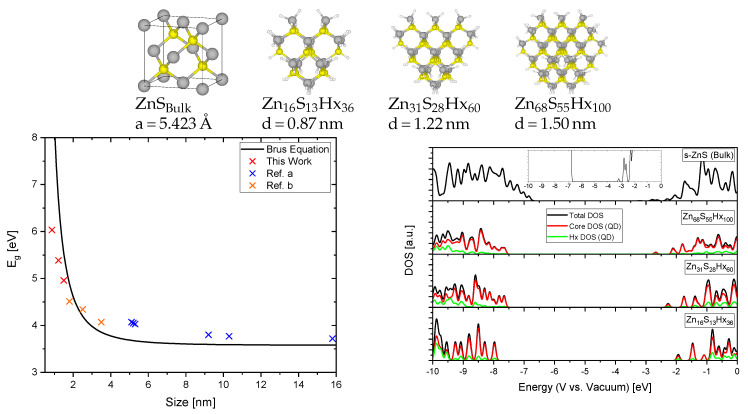
Relaxed structures of ZnS bulk and different sized ZnS quantum dots (QDs) (grey: Zn, yellow: S, white: Hx), the band gap energies based on our calculations, experiments and the Brus equation (**left**) and the density of states (DOS) with absolute energetic positions with respect to the vacuum (**right**). Reference a [40], Reference b [41].

Figure 6 shows the relaxed structures for s-ZnS bulk and QDs (passivated with fictional hydrogen atoms Hx to saturate dangling bonds on the surface atoms) with different sizes, the corresponding density of states (DOS) aligned to absolute energetic positions with respect to the vacuum and the energy band gaps E_g_ obtained by modeling, experiment and by theoretical calculations using the effective mass approximation (EMA, Equation (4), Brus equation [34]). Although the limitations of the EMA are well-known [35,36] the mismatch between experimental values, calculations based on the EMA and our calculations based on ab-initio DFT are minor. That shows that the values for the Hubbard U parameters elaborated above are transferable and suitable for both bulk and cluster systems.

(4) $$E_{g-\mathrm{QD}}=E_{g-\mathrm{Bulk}}+\frac{h^{2}}{8R^{2}}\left(\frac{1}{m_{e}}+\frac{1}{m_{h}}\right)-\frac{1.786e^{2}}{4\pi \epsilon_{0}\epsilon_{r}R^{2}}.$$

In Equation (4) $E_{g-\mathrm{QD}}$ and $E_{g-\mathrm{Bulk}}$ are representing the band gaps of the QD and the bulk material, *h* is Planck’s constant, *R* is the radius of the particle, $m_{e}$ = 0.34 $m_{0}$ and $m_{h}$ = 1.76 $m_{0}$ are the effective masses of excited electrons and holes of s-ZnS [37], *e* the electronic charge, $\epsilon_{0}$ the vacuum permittivity and $\epsilon_{r}$ = 8.3 the relative permittivity of s-ZnS [27].

The DOS calculation highlights the interesting change in the electronic structure of nanoparticles when compared to macroscopic materials. For nanometer sized semiconductor materials the band gap gets larger and also the band edge positions are being shifted. The CBM (or lowest unoccupied molecular orbital (LUMO) for QDs) redox potential is lowered and that of the VBM (or highest occupied molecular orbital (HOMO) for QDs) is increased. The reason for that is the so called quantum confinement effect [38]. This is observed when the radius of the particle becomes smaller than the exciton Bohr radius which results in motion of the electrons and holes spatially confined to the dimension of the QD (exciton Bohr radius of s-ZnS = 2.5 nm [39]). Similar to a particle in-a-box, which increases its energy when the box size decreases, the exciton will also increase in energy.

This leads to increased reductive and oxidative power of electrons and holes respectively that seem to further increase with decreasing particle size. That means that nanoparticles can catalyze reduction and oxidation reactions that can not proceed on the bulk material. To gain more insight in how internal interactions and synergetic effects in nanocomposites (consisting of semiconductor QDs and functionalized carbon support) can also alter such important electronic properties we studied the behaviour of ZnS QDs in different environments.

### 2.2. Fields and Defects

As already mentioned a functionalized carbon support such as oxidized CNTs can be electrostatically charged due to the introduction of carboxyl groups on its surface. Therefore, we investigated the behaviour of the electronic states of the QDs in the presence of electric fields. This was simulated by adding a saw-like potential to the bare ionic potential as implemented in the Quantum ESPRESSO package. It should be mentioned that electric fields induced by point charges, lines of charges and planes of charges also were simulated with the help of the Environ plug-in for Quantum ESPRESSO [42]. The outcome of these calculations followed all the same trend so for simplicity only the results of the electric fields simulated by saw-like potentials are presented.

**Figure 7:**
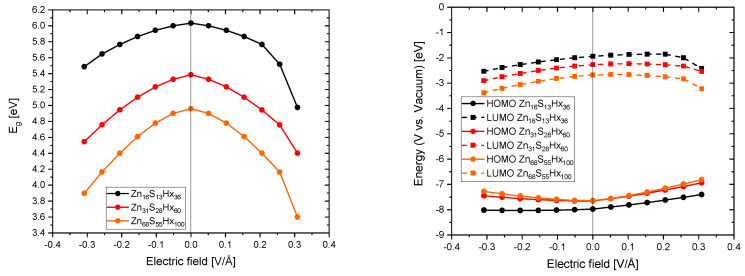
$E_{g}$ of different sized QDs (**left**) and the energetic position of the highest occupied molecular orbital (HOMO) and lowest occupied molecular orbital (LOMO) (**right**) as a function of the applied electrical field.

As it can be seen in Figure 7 an applied external electric field decreases the band gaps of the QDs independently of their size. Furthermore, the absolute energetic positions (with respect to the vacuum) of the HOMOs are increasing and that of the LUMOs are, for small positive electric fields increasing as well, but decrease for stronger ones. For the smallest simulated QD (Zn_16_S_13_Hx_36_) the increase of the LUMO is about ∼ 0.08 eV and gets even smaller with increasing particle size. Theoretically the reductive power of electrons could be boosted through this effect but practically it would be a minor improvement and it has to be considered that such small QDs have not been experimentally synthesized so far. Deducing from these findings the electric field that is induced by a functionalized carbon support would deteriorate the electronic properties (decreasing the band gap and in principle also the oxidative and reductive power of holes and electrons) of the QDs in nanocomposites.

**Figure 8:**
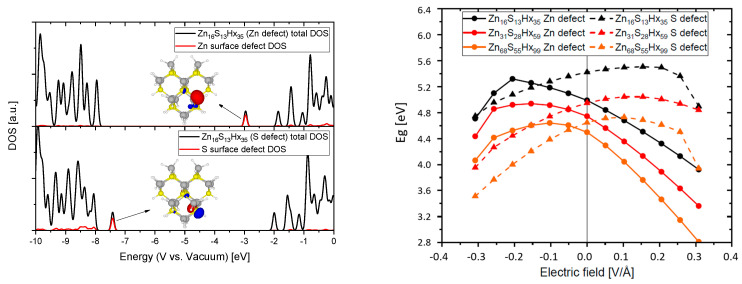
Density of states (DOS) and frontier orbitals of QDs with Zn and S surface defects (**left**) and the band gap $E_{g}$ of different sized QDs with surface defect states as a function of the applied electrical field (**right**).

Considering that catalytic redox reactions proceed at particle surfaces and their surface defect states that are not perfectly passivated like in our ideal simulations (with fictitious hydrogen atoms) we also investigated what influence an external electric field has on systems with surface defects. For that purpose we removed either at a zinc or a sulfur atom that is located at the surface one of the passivating atoms. The results of that approach are displayed in Figure 8. The DOS plots show that a dangling bond on a Zn surface atom leads to a defect state near the LUMO that can act as an electron trap whilst a dangling bond on a S surface atom leads to a defect state near the HOMO that can act as a hole trap. The |ψ|^2^ of these states is also illustrated (iso value = 0.005) indicating a local spread around the corresponding atom that does not reach far into the QD. Considering the previously elaborated depth by which electrical fields, exerted from functionalized CNTs, can reach into a QD (see Figure 2) it could fully overlap with the wave function of these trap states. An external field has great influence on the energetic position of these surface defects and therefore in contrast to the fully passivated case a bigger impact on the HOMO and LUMO which results in a more advantageous possibility of tuning the electronic properties. The band gap can be increased (up to 0.3 eV) and the absolute energetic position of the LUMO can be significantly increased (up to 0.7 eV, for the exact calculated values of the frontier orbitals see Figure A3) making it theoretically possible to considerably increase the reductive power of the material. Since in reality it is almost impossible to get rid of all surface defects it can be assumed that the synergetic effects arising from an induced electrical field in nanocomposites consisting of QDs and functionalized carbon support can be used to beneficially fine tune the electronic properties. As an example an increase of the CBM of ZnSe or ZnTe on this order of magnitude would enable an electron in such a material theoretically to reduce CO_2_ directly to its radical anion, see Figure 1. These findings are highlighting the great potential that the internal effects in nanocomposites exhibit to fine tune the electronic properties of its components and can be of substantial interest in the design of new photo- or electrocatalysts. Furthermore, these theoretical considerations can be supported by our experimental findings in which ZnS nanoparticles (synthesized with a Zn/S ratio > 1, suggesting a Zn rich surface) conjugated to oxidized CNTs (covered with negatively charged carboxyl groups on its surface, confirmed by zeta potential measurements) show an increased optical band gap (about ∼0.1 eV) when compared to the pristine ZnS nanoparticles [10].

### 2.3. Composites and Ligands

**Figure 9:**
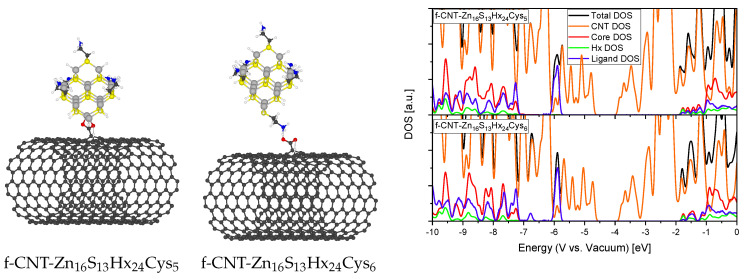
Relaxed structures of carboxylic functionalized CNT binding to Cysteamine terminated QDs (grey: Zn, yellow: S, white: Hx or H, black: C, red: O, blue: N) directly as passivating ligand (**left**) or electrostatically to the amine group of the Cysteamine (**middle**) and the corresponding DOS (**right**).

To research what influence the actual physical binding of the carbon support to the QD in nanocomposites exerts we investigated two different cases of a functionalized CNT (10,10) binding to a cysteamine terminated QD. The oxidized CNT was constructed by destroying two C-C bonds within a hexagon of the CNT structure. The resulting dangling bonds were saturated with oxygen and a hydroxyl group on one carbon atom to produce the carboxyl group and a hydrogen atom and a new covalent bond between two C atoms to form a pentagon to saturate the remaining dangling bonds [43]. The carboxyl group of the CNT is then considered to act as a passivating ligand itself in the first case and to bind electrostatically to the amine group of the cysteamine in the second case. The obtained relaxed structures and the corresponding DOS are illustrated in Figure 9. When the DOS of the functionalized CNT is compared to the pristine one (see Figure 5) it is observed that the oxidation and partial destruction of the CNT hexagonal structure leads to the loss of the metallic conducting character and the opening of a small band gap of E_g_ ∼ 0.7 eV. This is likely due to the hybridization of some carbon atoms from sp^2^ to sp^3^ which breaks the homogeneous delocalization of the π-electrons [44]. More in focus of this work were the electronic states of the semiconductor QD. No significant change of the nanocrystal core DOS between the two binding possibilities can be seen. Note that no surface defects were present in these systems since all dangling bonds on S and Zn atoms are terminated by either fictitious hydrogen atoms or organic ligands. Nevertheless, when compared to our previous calculation where the QD was passivated with only fictitious hydrogen atoms (see Figure 6) a shift of the band edge (HOMO, from −8.0 eV to −7.3 eV vs. vacuum) along with states arising from the ligand molecules near this band edge is noticed. These results suggest that the actual electrostatic binding of the oxidized CNT to the QD has no particular impact on the electronic structure of the semiconductor nanocrystal although it seems that surface passivating ligands can very well exert an influence on these properties. Therefore, we also investigated how different ligands modify the electronic structure of the QDs and how pronounced such influences are.

**Figure 10:**
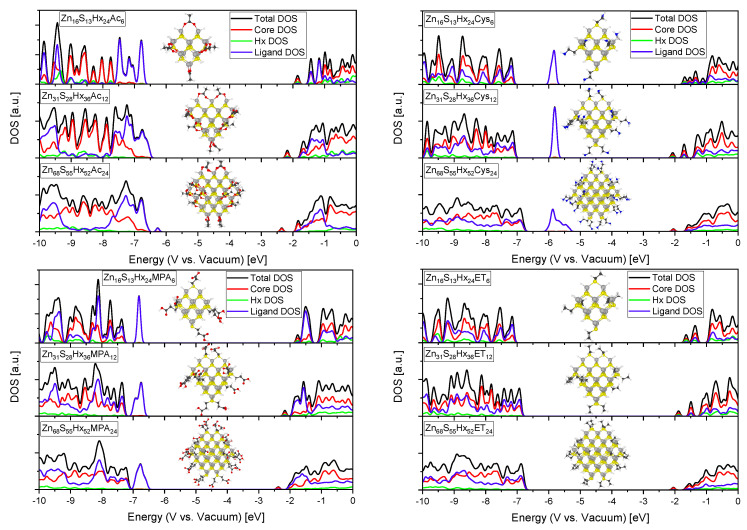
DOS of different sized QDs with various surface passivating ligands (grey: Zn, yellow: S, white: Hx or H, black: C, red: O, blue: N), Acetate (**top left**), Cysteamine (**top right**), 3-Mercaptopropionic acid (**bottom left**) and Ethanethiol (**bottom right**).

Commonly used thiol or carboxyl group containing molecules that are utilized in nanoparticle synthesis as stabilizers like cysteamine (Cys) [11], 3-mercaptopropionic acid (MPA) [45], ethanethiol (ET) and acetate (Ac) were modeled as capping ligands and compared (ET and Ac are used as computational inexpensive molecules that represent the influence of long chain thiols or fatty acids like 1-dodecanethiol [46], oleic acid [47] or stearic acid [48] that are often used in nanoparticle synthesis). Figure 10 shows the evolution of the computed DOS in ZnS nanocrystals passivated with different ligands. The figure clearly reflects the fact that surface passivating ligands are able to modify the electronic structure of nanocrystals. The contributions from the ligands to the total DOS is in differently sized QDs are comparable and show a similar trend. Despite it is obvious that various ligands have distinct DOS themselves and therefore different impact on the total DOS it is not straightforward that they also have an influence on the DOS of the QD core. For example, the LUMO which consists in all systems almost exclusively of contributions from the core QD can be shifted from −2.0 eV (Zn_68_S_55_Hx_52_ ET capped) to −2.4 eV (Zn_68_S_55_Hx_52_ Ac capped). These responses can be explained through ground-state QD-ligand interactions as the shape and energy of the wave functions of electrons and holes are sensitive to the finite potential barrier that is presented by the ligands that passivate the QD surface [49]. Such behaviour is also experimentally known as for example Kroupa et al. have shown that the band edges (and therefore the redox potential) of semiconductor nanocrystals can be greatly altered by modifying their surface properties by various stabilizing ligands [50]. Moreover, the results show that anchoring or additional functional groups of the ligands can introduce hole traps near the HOMO (as it can be observed for Ac, Cys and MPA). It was also tested if the alkyl chain length of the ligand has an impact but as it can be seen in Figure A4 that the differences are negligible allowing the conclusion that mainly the anchoring group and additional functional groups of the ligand determine the barrier potential that the ligand represents. Nevertheless, it should be mentioned that all these calculations do not provide any information about possible charge transfers between the ligands and the QDs and also no information about absorption coefficients so that experimentally obtained optical band gaps and redox potentials could differ. As an example Gabka et al. showed that ligands with different binding anchor groups can significantly affect the optical absorption behaviour [51].

### 2.4. Comparison with Experimental Results

Coming back to the experiments as displayed in Figure 3, from the calculations discussed above it now becomes clear, that simple and comparably loose electrostatic binding of well-formed and ligand- passivated QDs to a charged carbon support has a minor influence on the band gap and band edges of the QD. Surface defects and their interaction with the environment are much more important, and therefore we see, that direct growth of the QDs on a (in the experimental case multi-walled) functionalized CNT without ligand spacers will have a much larger impact–the combined action of surface/interface defects and electrostatic fields in direct neighbourhood has the potential to push the band edges to higher potentials, visible in the shift of excitation onset shifted by about 0.1 eV to higher energies. Considering the role of ligands, the main influence on the optical and electronic properties is based on the chemistry of the head group, since it will either cause additional defects or saturate existing ones. The length of the tail group is of minor importance in this context and will rather be important when discussing the colloidal properties.

## 3. Materials and Methods

### 3.1. Synthesis and Characterization

All materials (quantum dots with ligands, functionalized carbon nanotubes, heteroaggregates and composites) were synthesized by standard wet chemical methods and characterized by standard procedures, as described in detail in prior work [10,11] and references therein. Typical quantum dots are charged positively, whereas the CNTs due to oxygen-dominated surface groups are exhibiting negative charge.

### 3.2. Computational Details

**Figure A1:**
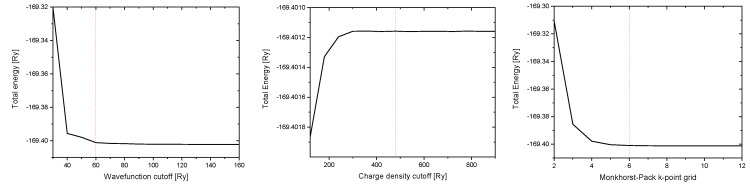
Convergence tests of the kinetic cutoff for wavefunctions (**left**), the kinetic cutoff for the charge density (**middle**) and the Monkhorst-Pack k-point grid (**right**).

**Figure A2:**
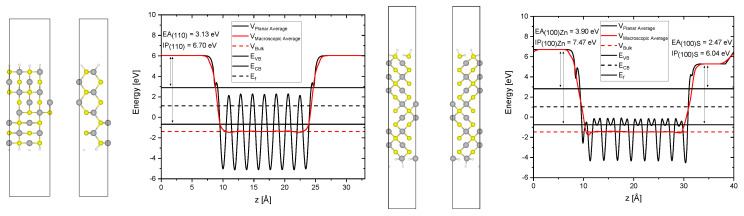
View along different axes along the unit cell of for the (110) (**left**) and (100) (**right**) surface slab calculation and the corresponding calculated planar and macroscopic average electrostatic potential.

**Figure A3:**
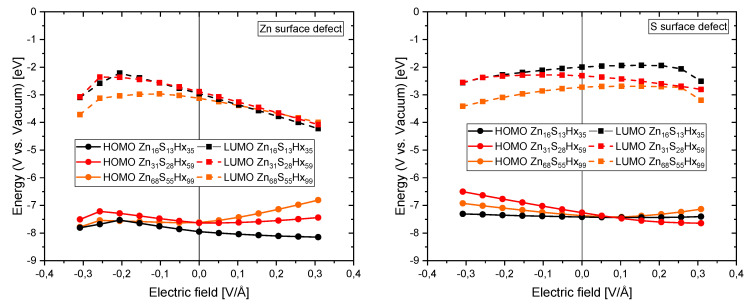
Absolute energetic position of the HOMOs and LUMOs for different sized QDs with Zn (**left**) or S (**right**) surface defects as a function of the applied electrical field.

**Figure A4:**
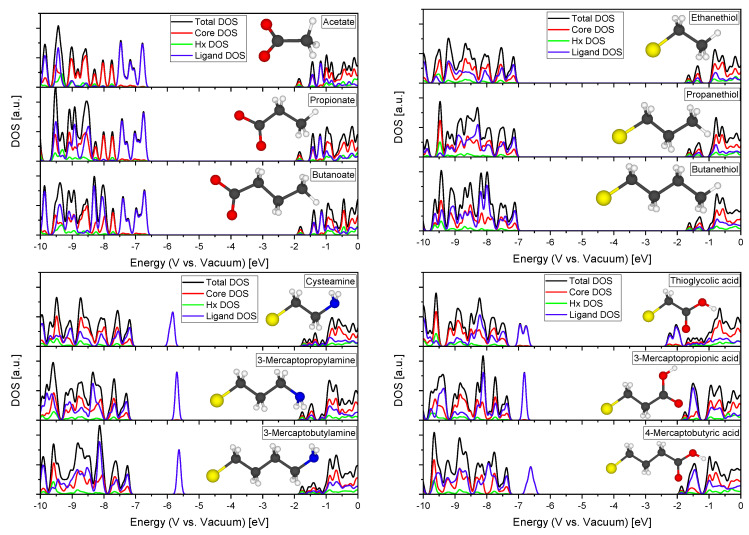
DOS of Zn_16_S_13_Hx_24_ QDs with different surface passivating ligands (yellow: S, white: H, black: C, red: O, blue: N).

Calculations were performed in the framework of an ab initio density functional theory (DFT) computational method [52,53], as implemented in the Quantum ESPRESSO package [54,55]. Energies were calculated by using a plane wave basis set, scalar relativistic ultrasoft pseudopotentials (USPP) and the generalized gradient approximation (GGA) with the Perdew-Burke-Ernzerhof (PBE) functional to describe the exchange-correlation potential [56]. To improve the accuracy of the calculations a Hubbard U correction [57] was applied on the Zn 3d and S 2p states to correct the well known problem that LDA and GGA functionals underestimate the band gap and are not able to reproduce the correct position of *d* or *f* states of transition or rare earth metals [58]. Since the linear-response theory (used to calculate ab initio values for the Hubbard U parameters [59]) has its limitations and fails for closed-shell systems [60] the U parameters for Zn-d and S-p were optimized simultaneously in order to reproduce the experimental lattice parameter, band gap and the relative position of the Zn 3d states of sphalerite bulk structure ZnS (s-ZnS) similar to Mattioli et al. and Khan et al. [61,62]. Satisfactorily converged results were achieved by using 60 Ry as the kinetic cutoff for wave functions and 480 Ry as kinetic energy cutoff for the charge density and potential (see Figure A1). The convergence thresholds for self consistency, the total energy, the forces for ionic minimization and the pressure for variable cell relaxations were set at 1.0 × 10${}^{-8}$ Ry, 1.0 × 10${}^{-4}$ Ry, 1.0 × 10${}^{-3}$ Ry/Bohr and 5.0 × 10${}^{-4}$ GPa respectively. A 6 × 6 × 6 and 1 × 1 × 12 Monkhorst-Pack k-point grid including the Γ point was used for sampling the 1st Brillouin zone of bulk s-ZnS and pristine CNTs while a Γ point only sampling was used for the calculation of the stand alone QDs and the composites consisting of CNTs and QDs. The supercell with the QDs or composites located at the center contains a vacuum region of at least 10 Å to avoid periodic interactions. Furthermore, to compensate surface defect states arising from dangling bonds on surface ions these dangling bonds were passivated by pseudoatoms with fractional nuclear charges (1.5 for Zn^2+^ and 0.5 for S${}^{2-}$) as suggested by Huang et al. [63]. In the zincblende bulk structure each Zn atom is surrounded by four S atoms and vice versa. The formal valence of Zn atoms is two and for S atoms is six. So 0.5 e of each Zn and 1.5 e of each S atom are provided to form a single Zn-S bond. Therefore fictitious hydrogen atoms with a fractional nuclear charge of 1.5 (for a dangling bond on a Zn atom) and 0.5 (for a dangling bond on a S atom) were used to saturate the atoms exposed on the QD surface respectively. Electronic band and state positions with respect to the vacuum level were obtained by calculating the planar average electrostatic potentials along the z-axis and the vacuum region is used as the absolute energy reference. For the ZnS bulk system the whole space is filled with periodic crystals and such a reference cannot be defined. In that case a methodology that involves both bulk and a surface/slab calculation is used and the vacuum level is then defined far away from the surface in the vacuum region and the bulk average electrostatic potential as the value of the macroscopic averaged electrostatic potential in the middle of the slab (see Figure A2). The QDs were constructed by cutting out a spherical shape from the optimized underlying zincblende bulk structure by selecting all atoms within a radius of r < x^2^ + y^2^ + z^2^ around a sulfur or zinc atom. This leads to non-stiochiometric QDs that were necessary to ensure charge neutrality when charged ligands were present. All systems were optimized with respect to their geometry before any electronic structure calculation was performed.

## 4. Conclusions

In this report it has been shown, that properly adjusted and calibrated standard DFT calculations are capable to describe and predict the influence of interface defects, passivating ligands and electrostatic fields on the optical and electronic properties of nanocomposites. The practically important case of ZnS in contact with carbon was chosen as example and it can be concluded, that the combination of local electrostatic fields and surface defects will offer the possibility to fine tune the properties of potential photo- and electrocatalysts. Overall the combined investigation of surface passivating ligands and external fields suggest that an ingenious combination of a charged carbon support such as oxidized CNTs and QDs with ligands that regulate the distance and therefore the electrical field that the QD feels, can be utilized to modify and change the electronic properties at will.

## Figures and Tables

**Table 1:** Comparison of generalized gradient approximation (GGA), GGA+U and experimental results for ZnS.

Method	$E_{g}$ [eV]	${\mathrm{Ed}}_{3/2}$ [eV]	${\mathrm{Ed}}_{5/2}$ [eV]	*a* [Å]	EA_110_ [eV]	IP_110_ [eV]
GGA	2.03	6.25	5.76	5.435	3.33	5.36
GGA+U	3.57	9.37	8.86	5.423	3.13	6.70
Exp.	3.6 [26]	9.38 [27]	8.82 [27]	5.42 [28]	3.9 [29]	7.5 [29]

## Appendix A

In Figure A1 the convergence tests for the kinetic cutoff for wave functions and charge density and the Monkhorst-Pack k-point grid for bulk s-ZnS are shown. The chosen values for these parameters are highlighted by the red dashed lines (at 60 Ry, 480 Ry and a 6 × 6 × 6 Monkhorst-Pack k-point grid including the Γ point). These values also correspond to converged results of the pristine CNTs (except for the usage of a 1 × 1 × 12 Monkhorst-Pack k-point grid), the QDs and the composites consisting of CNTs and QDs (Γ only k-point).

In Figure A2 the planar and macroscopic average electrostatic potential along the z-axis of the (110) and (100) crystal plane slab calculation is illustrated. It is referenced to the macroscopic average electrostatic potential of the bulk material in order to obtain the absolute energetic positions of the valence and conduction band with respect to the vacuum. Physically these values represent the ionization potential (IP) and electron affinity (EA). It should be mentioned that for the (100) surface a dipole correction with the length of 5% of the unit cells z-axis is used. This can be seen as the energy in the vacuum region increases there linearly. That was necessary since this surface has polar terminations (either zinc or sulfur terminated) that lead to a non-vanishing dipole density in the vacuum region (capacitor effect).

The absolute energetic positions of the frontier orbitals with respect to the vacuum for three different sized QDs with either an unpassivated Zn- (left) or S atom (right) on its surface as a function of the electrical field is displayed in Figure A3. As it can be seen the influence of the electrical field is more pronounced than for fully passivated QDs (compare with Figure 7).

Displayed in Figure A4 are the DOS of a Zn_16_S_13_Hx_24_ QD with different functional surface passivating ligands. It reveals that the alkylchain length has an almost negligible effect on the DOS allowing the conclusion that mainly the anchoring and functional groups of surface passivating ligands play a role in the modification of the electronic structure of semiconductor nanocrystals. This also validates the usage of Acetate and Ethanethiol as computational inexpensive molecules to represent the influence of long chain thiols or fatty acids. Note that the included images show only the ligand that was used (deprotonated at the anchoring group that binds to the QD surface), not the system of the passivated QD that was actually calculated here.
